# Lower oxygen saturation with higher rates of norepinephrine in bone fractures of polytrauma patients: a pilot study

**DOI:** 10.1186/s13054-023-04657-6

**Published:** 2023-09-25

**Authors:** Laura Koch, Marcel Orth, Tobias Fink, Andreas Meiser, Thomas Volk, Michael D. Menger, Matthias W. Laschke, Torsten Pastor, Matthias Knobe, Tim Pohlemann, Bergita Ganse

**Affiliations:** 1 Werner Siemens-Endowed Chair for Innovative Implant Development (Fracture Healing), Departments and Institutes of Surgery, Saarland University, Homburg, Germany; 2 Department of Trauma, Hand and Reconstructive Surgery, Departments and Institutes of Surgery, Saarland University, Homburg, Germany; 3 Department of Anaesthesiology, Intensive Care and Pain Therapy, Saarland University, Homburg, Germany; 4 Institute for Clinical and Experimental Surgery, Saarland University, Homburg, Germany; 5 Department of Orthopaedic and Trauma Surgery, Lucerne Cantonal Hospital, Lucerne, Switzerland; 6 Department of Orthopaedic and Trauma Surgery, Westmuensterland Hospital, Ahaus, Germany; 7 Medical Faculty, University of Zurich, Zurich, Switzerland; 8 Medical Faculty, RWTH University Aachen, Aachen, Germany

**Keywords:** Multiple trauma, Catecholamines, Noradrenaline, Non-union, Fracture healing, Microcirculation, Perfusion, O2C, Injury, Mean arterial pressure

*Trial registration*: The study is registered in the German Clinical Trials Register (DRKS00031942)

Hypoxia is a risk factor for non-union and appears to be one of the causes of the higher incidence of non-union in patients with multiple injuries [1]. Norepinephrine (NE) decreased bone perfusion in animal studies [2]. While the intramedullary bone-marrow pressure usually correlates with the systemic blood pressure, the contrary was observed under NE treatment: the systemic blood pressure increased, and simultaneously, the intramedullary bone-marrow pressure dropped [2]. This effect was independent of the systemic blood pressure and is probably caused by α-receptor-transmitted vasoconstriction of the nutrient vessels. Patients in a more severe state receive NE to increase their systemic blood pressure. The target systolic blood pressure, according to current guidelines, is 80–90 mmHg [3]. NE is currently the most frequently used and recommended catecholamine for polytraumatized patients in intensive care units [3, 4]. In human patients, the effect of *i.v.*-NE administration on the blood perfusion in fractures and the correlation with the systemic arterial blood pressure have not yet been investigated. We hypothesized lower oxygen saturation (SO_2_) in the fracture gap with higher doses of NE, independent of MAP.

In a pilot trial, we used the laser-Doppler device ‘Oxygen to see’ (O2C, LEA Medizintechnik, Winchesterstr. 2, D-35394 Gießen, Germany) to measure SO_2_, haemoglobin (Hb), and blood flow (BF) in 3, 10, and 14/16 mm depth in four tibial and clavicle fractures of three patients with multiple injuries who received NE. The O2C operates with a laser (wavelength 820 nm) and a detector in the white-light spectrum range (wavelength 500–800 nm) [5]. Probes were covered in single-use ‘Ultracover for TEE Endocavity Probe Cover’ (ECOLAB, Microtek Medical B.V., Hekkehorst 24, 7207 NL-Zutphen, The Netherlands), 25 × 11 × 1000 mm, made of polyurethane. The non-invasive probe was fixed on the skin with black kinesiology tape to avoid measurement error by the ambient light and to standardize the contact pressure. In each of the fractures, multiple measurements were conducted throughout the time course of treatment with varying NE rates. During each measurement, three recordings of 10 s each were taken in slightly different spots over each fracture gap and averaged. MAP was measured by an arterial probe as part of regular patient monitoring and recorded for each measurement. Of note, the present study was purely observational and did not interfere with the NE administration rate. NE was administered through injection pumps solely operated by the intensive care unit staff independent of the study. Multiple linear regression statistics were conducted with forced entry for each of the perfusion parameters and each depth separate as the dependent variable. The relationships of each of these parameters with the NE rate and the MAP as independent variables were explored.

Three male patients with three tibial fractures and a clavicle fracture were included (age 57.3 ± 33.1 years, weight 77.7 ± 6.8 kg, height 177.7 ± 4.0 cm, BMI 24.7 ± 3.1 kg/cm^2^). A total of 125 measurements were taken, all within the first week after fracture. Significantly higher NE rates were associated with lower SO_2_ in 10 mm (*p* = 0.018) and 14/16 mm depth (*p* = 0.006) of fractures (Fig. 1A–C). Significant correlations between the MAP and SO_2_ were found in 3 (*p* = 0.007) and 10 mm depth (*p* = 0.030) (Fig. 1D–F). Higher NE administration rates and lower MAP were associated with lower SO_2_. Hb and BF in the fracture gap were neither affected by the NE rate nor the MAP. In 10 mm depth, up to 16.2% (adjusted R^2^) of the variability of SO_2_ could be explained by NE and MAP. In 3 mm, up to 14.7% of the variability could be explained by MAP alone, and in 14/16 mm, up to 13.5% by NE alone. Thus, only NE and not MAP had an effect on SO_2_ in 14/16 mm depth inside the fractures.

**Fig. 1:**
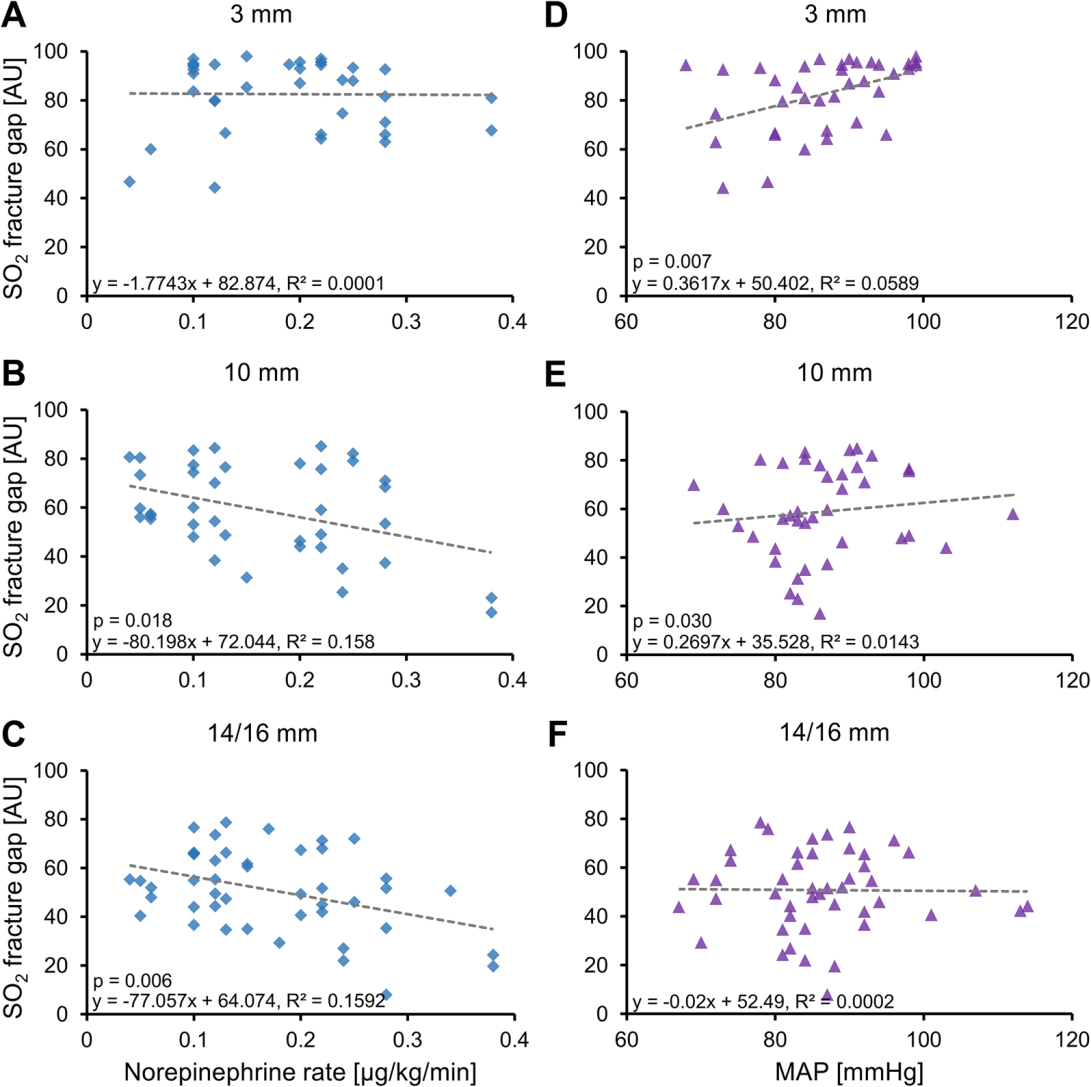
Relationships of the NE rate (**A**–**C**) and the MAP (**D**–**F**) with the measured SO_2_ values. *P* values from the multiple linear regression analysis are shown only, if significant

The present pilot study is the first to measure fracture perfusion in patients with NE. The presented findings need to be confirmed in larger cohorts. The reported data indicate an association of NE administration rates with decreases in bone fracture oxygenation that may be caused by the NE, but could also be related to other mechanisms associated with the more severe state of the patients that require NE treatment. As lower SO_2_ in fractures may lead to delayed bone healing and non-union, it might be of interest to study the effects of alternative drugs for blood pressure elevation on bone perfusion that do not show a vasoconstrictive effect on bone. Among the catecholamines, dobutamine mainly has an effect on β_1_ receptors, but shows almost no α-receptor-mediated vasoconstriction. If it can be confirmed that NE decreases bone blood supply, NE might be used to decrease blood loss from fractures. Laser-Doppler-based devices and other instruments should be validated for perfusion measurements and SO_2_-monitoring in bone fractures of patients.

## Data Availability

The data set obtained in the current study is available from the corresponding author on reasonable request.

## Abbreviations

BF: Blood flow
Hb: Haemoglobin
*i.v.*: Intravenous
MAP: Mean arterial pressure
mmHg: Millimetre of mercury
NE: Norepinephrine
O2C: Oxygen to see (device)
SO_2_: Oxygen saturation
